# Volatile Profile of 16 Unifloral Pollen Taxa Collected by Honey Bees (*Apis mellifera* L.)

**DOI:** 10.3390/insects16070668

**Published:** 2025-06-26

**Authors:** Vasilios Liolios, Chrysoula Tananaki, Dimitrios Kanelis, Maria Anna Rodopoulou, Fotini Papadopoulou

**Affiliations:** Laboratory of Apiculture-Sericulture, Aristotle University of Thessaloniki, 57001 Thessaloniki, Greece; dkanelis@agro.auth.gr (D.K.); rodopoum@agro.auth.gr (M.A.R.); foteinpi@agro.auth.gr (F.P.)

**Keywords:** bee pollen, volatile organic compounds, pollination, honey bees’ preference, GC-MS

## Abstract

Bee pollen’s aroma combined with other floral components serve various purposes, including attracting pollinators and signaling the availability of food sources. The present study aimed to comparatively analyze the volatile profiles of unifloral pollen taxa. Bee pollen loads were collected using pollen traps and sorted based on their botanical origin, determined by color and pollen grain morphology. The separated pollen samples were analyzed using a Purge & Trap/GC-MS system, identifying the volatile profiles of pollen from 16 plant species. The analysis revealed distinguished differences in the total volatile organic compounds (VOCs) among the various pollen species. Notably, the pollen from *Erica manipuliflora*, *Papaver rhoeas*, and *Sisymbrium irio* contained the highest number of VOCs, with 54, 51, and 42 substances detected, respectively. Certain volatile compounds appeared to correlate with increased bee visitation. For instance, 4-methyl-5-nonanone was uniquely found in *E. manipuliflora* pollen, while isothiocyanate compounds were exclusively present in species of the Brassicaceae family. Therefore, given the significant impact of VOCs on honey bees’ preferences, it is essential to consider not only the nutritional value of bee pollen when evaluating its beekeeping value, but also its aroma profile.

## Simple Summary

The aroma of a flower is composed of a mixture of numerous volatile compounds. Various floral organs contribute to this aroma, including the anthers and the pollen they produce. Although it is often assumed that pollen scent influences honey bee preferences, research on insect attraction to bee pollen aromas is limited and has not extensively focused on unifloral pollen species. In this study, the volatile profiles of 16 unifloral bee pollen taxa with significant importance to beekeeping were analyzed, re-vealing differences in their volatile compound profiles. Certain volatile compounds, such as isothiocyanate compounds found in the pollen of the Brassicaceae family, may be associated with higher bee visitation rates. This suggests a potential explanation for honey bees’ behavior during pollen collection and their preference for specific pollen types.

## 1. Introduction

Plants synthesize and emit a large variety of biogenic volatile organic compounds (VOCs), thereby interacting dynamically with their immediate environment. These compounds include fatty acid derivatives, terpenoids, phenylpropanoids/benzenoids, and amino acid derivatives [1,2]. The emission of certain VOCs into the atmosphere can have profound biological effects on plant interactions with other organisms, as well as environmental consequences, by influencing the physicochemical properties of the atmosphere [3,4].

Floral volatiles are typically emitted from petals, although other floral or vegetative tissues may also contribute significantly to their production and release [5]. Indeed, Dudareva & Pichersky [6] reported the emission of VOCs from non-floral vegetative tissues as well. Nevertheless, some compounds are exclusively produced by floral organs, serving functions such as the attraction of mutualistic visitors or the deterrence of antagonists. In addition to petals, other floral components such as sepals, nectar, and pollen are also involved in the emission of scent mixtures [7,8,9,10]. In certain species, the androecium—the male reproductive structures of the flower, including stamens, anthers, and pollen grains—is hypothesized to produce distinctive volatile compounds involved in pollinator attraction [11].

The production of VOCs in plants serves a variety of ecological functions. Repellent chemicals—primarily terpenoids—constitute a fundamental defense mechanism that deters harmful organisms while mitigating adverse environmental conditions [12,13]. Benzenoids, however, are encountered with equal frequency [14] and, in some cases, in greater abundance than terpenoids [15]. It is also known that plants compete with one another for optimal growth conditions. According to Rasher et al. [16], the evolution of allelopathic compounds has been crucial in enabling plants to cope with the intense competition that arises under dense planting or crowding. Nonetheless, the most significant ecological role of VOCs is the attraction of suitable pollinators [2], especially considering that 85% of plant species rely on animal pollinators for successful reproduction [17]. Chittka & Raine [18] described the diversity of flowering plants as a “biological market,” from which pollinators select species that offer the greatest rewards. Thus, specific chemical compounds play a key role in facilitating botanical recognition by pollinators. This intricate plant–pollinator interaction is further supported by observations from Negre et al. [19], who reported a decline or alteration in floral VOC emission following successful pollination in some species. Such changes likely serve to reduce unnecessary visitation by floral visitors, especially those that could damage fertilized flowers, while promoting efficient foraging toward unpollinated flowers [20].

Regarding pollen, the characterization of its scent-related chemical components remains limited, mainly due to the methodological challenges in sampling and analyzing pollen volatiles [21]. Studies of VOCs in the pollen of angiosperms using headspace extraction techniques have confirmed the presence of distinctive olfactory profiles, chemically distinct from those of other floral parts [22,23]. Plants with pollen of a characteristic and intense odor confer an advantage by directly signaling the presence of rewards to potential pollinators, thereby enhancing the plant’s competitiveness within floral communities. Doull [24] was the first to demonstrate the ability of honey bees to distinguish between pollen odors of different plant taxa. Pollen scent appears to play an important role in pollinator attraction and tends to be more pronounced in entomophilous species [25], which rely predominantly on insect pollination. Knoll [26] was the first to investigate the origin of pollen scent, attributing it primarily to the outer lipid-rich layer (pollenkitt) of the pollen grains, which constitutes approximately 5% of their mass [25]. Additionally, Dobson et al. [27] suggested that some VOCs emitted from petals may be adsorbed onto pollen grains from the surrounding air.

However, the plant–pollinator relationship is bidirectional. Therefore, understanding the behavior and response of pollinators to pollen-derived volatile compounds is equally important. Several studies have confirmed that honey bees [25,28], beetles [29,30], and hoverflies [31] are attracted to odors during foraging. According to Lunau [32], bumblebees combine visual (e.g., anther color) and olfactory (e.g., pollen odor) cues to select flowers for visitation. Dobson et al. [28], in experiments with *Rosa rugosa* pollen, demonstrated that bumblebees are capable of distinguishing between flowers with different pollen quantities based on olfactory cues.

Honey bees collect pollen from a wide range of plant species, both cultivated and wild [33]. Although several studies suggest that the sensory characteristics of bee pollen vary significantly among plant species [34,35], available data on the volatile composition of pollen from individual plant species remain limited [36,37,38]. To date, most research has focused on the volatile profiles of mixed pollen samples [39,40,41,42,43,44].

Considering all the above, the aim of the present study was to comparatively assess the volatile profiles of unifloral pollen samples. The presence of characteristic volatile compounds in certain pollen types may be associated with their increased visitation by honey bees. Consequently, when evaluating the beekeeping value of a pollen-producing plant, its volatile profile should be considered alongside its chemical composition and nutritional value, due to its potential influence on pollen attractiveness.

## 2. Materials and Methods

### 2.1. Sample Collection, Separation, and Pollen Identification

A total of 16 unifloral pollen species were analyzed, which were collected from the experimental apiary of the Laboratory of Apiculture-Sericulture (A.U.TH.) using outdoor front pollen traps. The separation of pollen loads was carried out as promptly as possible after their collection from the pollen tray to minimize any potential contamination with volatile compounds. The separation process was primarily based on color, shape, and size. The separated samples were examined for purity and botanical origin using an optical microscope (Olympus, B X 40, Tokyo, Japan) with 400× magnification. Purity was assessed by calculating the percentage of the dominant pollen species present in 1 g of each sample. All samples exhibited purities greater than 95%, which exceeds the threshold recommended by Campos et al. [45] for confirming uniflorality (>80% of pollen grains from a single botanical species). Pollen analysis was performed using the method described by Louveaux et al. [46,47]. Specifically, the 16 unifloral pollen taxa whose volatile profile was studied were as follows: *Acer* sp. L. (Sapindaceae), *Actinidia chinensis* Planch. (Actinidaceae), *Brassica napus* L. (Brassicaceae), *Castanea sativa* Mill. (Fagaceae), *Cichorium intybus* L. (Asteraceae), *Cistus* sp. L. (Cistaceae), *Erica manipuliflora* Salisb. (Ericaceae), *Lamium* sp. L. (Lamiaceae), *Parthenocissus inserta* (A.Kern.) Fritsch (Vitaceae), *Papaver rhoeas* L. (Papaveraceae), *Pinus halepensis* Mill. (Pinaceae), *Ranunculus* sp. L. (Ranunculaceae), *Rubus* sp. L. (Rosaceae), *Sisymbrium irio* L. (Brassicaceae), *Trifolium* sp. Tourn. ex L. (Fabaceae), and *Verbascum* sp. L. (Scrophulariaceae).

### 2.2. Isolation and Identification of Volatile Organic Compounds

The process of isolating and identifying volatile organic compounds involves the following stages for each sample.

#### 2.2.1. Sample Preparation

A 2 g sample of pollen was accurately weighed to ±0.001 g, diluted with 13 g of ultrapure water (Millipore Simplicity 185 system, Merck Millipore, Darmstadt, Germany), and the solution was transferred into a 25 mL glass vial of a Purge and Trap extraction system (OI Analytical, model 4560, New York, NY, USA). The extraction system was equipped with a heating capability for the sample and an automatic sampler. The mixture was subjected to mechanical stirring (vortex) for 30–60 s. To prevent foam formation, a 40 cm stainless steel (No. 316) spiral coil was used.

#### 2.2.2. Sample Extraction

The sample was at first heated at a low temperature (40 °C) for 2 min without gas flow to reduce viscosity, facilitating the easier passage of helium (He) gas through the solution during extraction. Then, the volatile compounds were isolated by passing high-purity He (99.999%) gas at a flow rate of 40 mL/min through the glass vial for 40 min. The sample temperature was maintained at 40 °C throughout this process. During extraction, the volatile and semi-volatile components of the sample were trapped in a Tenax 07 column (Buchem B.V., Apeldoorn, The Netherlands). Following extraction, moisture was removed from the trap by heating to 100 °C for 2 min. The trapped compounds were released by heating the trap to 180 °C for 6 min. To prepare the trap for the next sample, it was heated for 7 min at 200 °C to ensure complete cleaning.

#### 2.2.3. GC Analysis

After extraction, the compounds were transferred to the gas chromatograph (Agilent, model 6890, Agilent Technologies, Santa Clara, CA, USA) via a thermostatted (100 °C) transfer line. The sample was introduced into the column using a split-splitless injector at 220 °C. The separation of the components was achieved on a capillary HP-5MS column, CA, USA (30 × 0.25 mm, df = 0.25 μm), using the following temperature program: 40 °C (hold for 5 min), ramped at 10 °C/min to 55 °C, ramped at 30 °C/min to 120 °C, ramped at 100 °C/min to 230 °C, and ramped at 200 °C/min to 280 °C (hold for 5 min). The carrier gas flow (He) was set to 1 mL/min. Detection of the separated compounds was carried out using a mass spectrometer (Agilent, model 5973, Agilent Technologies, CA, USA), operating under the following conditions: interface temperature 280 °C, ionization source temperature 230 °C, quadrupole temperature 150 °C, and ionization voltage +70 eV. Chromatographic data were processed and analyzed using MSD ChemStation software (5973N, version G1701DA). Peak identification was performed by comparing the mass spectra to those stored in the NIST05 electronic library. Additionally, previously established identification tables (retention times and spectra) for volatile compounds were consulted [48]. The percentage participation was estimated by dividing the peak area of the isolated volatile compound by the sum of the peak areas of all compounds detected in the sample.

## 3. Results

A total of 140 VOCs were detected from the analysis of 16 unifloral pollen samples, of which 117 were successfully identified and 23 remained unknown. Peaks exhibiting low spectral match scores were not identified and are herein referred to as “unknown,” with their corresponding mass fragments described. Additionally, 12 compounds were identified at the isomeric level. The complete list of volatile compounds, ordered sequentially based on their retention time (RT), is presented in Table 1. Due to the complexity of certain compound names and the difficulty in referring to them throughout the text, a coding system was implemented. These codes are listed in the second column of Table 1. For VOCs of particular importance, the corresponding chemical names are also provided. In some cases, alongside the IUPAC name, commonly used trivial names are included—especially for constituents typically found in essential oils, where such names are more widely recognized. The mass spectral fragments for each VOC are reported in parentheses, with the base peak indicated by underlining.

Among the 140 VOCs identified across all samples, only seven were consistently detected in 100% of the analyzed unifloral pollen types. Of these, four were aldehydes—furfural (C28), heptanal (C49), nonanal (C111), and decanal (C126)—while the remaining three were hydrocarbons—toluene (C17), ethylbenzene (C34), and α-pinene (C52). Additionally, two VOCs were present in 94% of the samples, being absent in only one pollen type each. Specifically, octanal (C78) was not detected in *Pinus* sp., whereas 2-ethyl-1-hexanol (C85) was absent from *Acer* sp.

**Figure 1:**
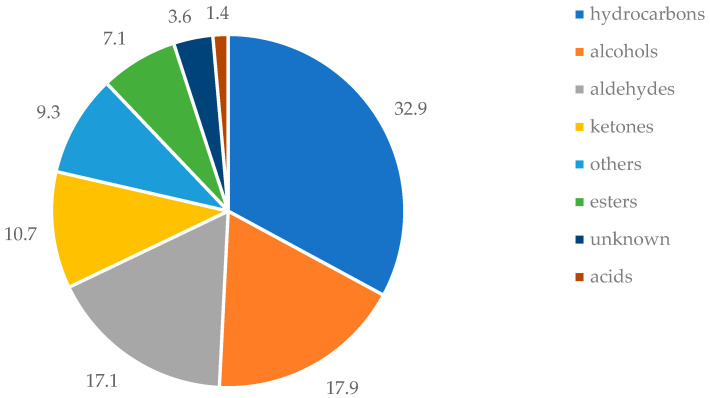
Percentage contribution of different classes of VOCs to the profile of unifloral pollen types.

The pronounced presence of hydrocarbons, aldehydes, and alcohols is further illustrated in Figure 1, which presents the relative abundance of major chemical classes detected across the unifloral pollen samples.

Hydrocarbons represented the most prevalent class, comprising 32.9% of all identified VOCs. Alcohols and aldehydes followed with similar contributions, accounting for 17.9% and 17.1%, respectively. Ketones (10.7%), esters (7.1%), and carboxylic acids (1.4%) were detected at lower frequencies. A minor proportion of the compounds (3.6%) could not be structurally identified and were designated as unknowns. An additional 9.3% encompassed structurally diverse and infrequently occurring compounds—including heterocycles, sulfides, and isothiocyanates—which, due to their chemical heterogeneity and sporadic detection, were collectively classified under a miscellaneous group. Among the 16 unifloral pollen samples analyzed, those from *E. manipuliflora*, *P. rhoeas*, and *S. irio* exhibited the greatest chemical complexity, with 54, 51, and 42 VOCs detected, respectively. In contrast, the lowest VOC richness was observed in *Pinus* sp. and *Lamium* sp., each yielding only 25 identified VOCs, while the VOC profile of *Acer* sp. was notably limited, with just 27 compounds detected.

**Figure 2:**
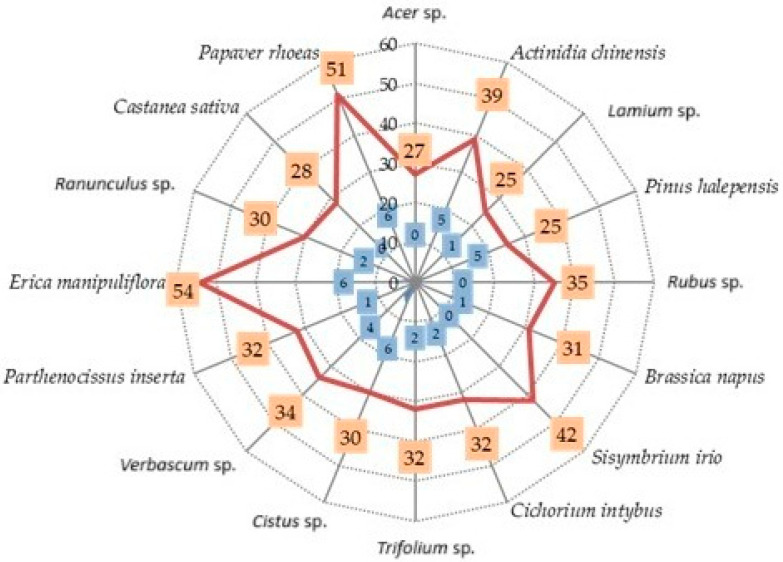
Total number of VOCs 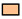 and number of unique VOCs 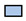 identified in the examined unifloral pollen samples.

The total number of VOCs identified in each pollen sample (orange rectangles), alongside the number of VOCs found uniquely in each unifloral pollen taxa analyzed (blue rectangles), is given in Figure 2.

Beyond the total number of volatiles detected, the presence of species-specific VOCs may be associated with the attractiveness of individual pollen types. The highest number of such unique compounds was identified in the pollen of *P. rhoeas*, *Cistus* sp., and *E. manipuliflora* (six unique VOCs each), followed by *A. chinensis* and *P. halepensis*, each with five exclusive compounds.

A detailed overview of these species-specific volatiles, along with their percentage participation in each pollen type, is presented in Table 2.

The majority of the characteristic compounds were detected in relatively low concentrations, typically under 5%. However, certain volatile compounds were found to have significantly higher relative abundances in specific pollen types. These compounds were classified as “major” not due to their exclusive presence in a particular species, but rather because their percentage participation was notably higher compared to other compounds identified in the same pollen samples.

A detailed list of these major compounds, along with their respective concentrations, is provided in Table 3.

Characteristic examples include the presence of octane (C23) at 67.90% in *C. sativa* pollen, 29.31% in *Lamium* sp. pollen, and 26.79% in *Acer* sp. pollen, 6-methyl-5-hepten-2-one (C70) at 61.99% in *Verbascum* sp. pollen, 4-isothiocyanato-1-butyne (C71) at 58.45% in *S. irio* pollen, 4-ethyl-5-methylthiazole (C102) at 54.52% in *B. napus* pollen, hexanal (C24) at 47.16% in *P. inserta* pollen, 2-ethyl-1-hexanol (C85) at 39.96% in *Cistus* sp. pollen, β-pinene (C64) at 38.42% in *Pinus* sp. pollen, 6-methyl-5-hepten-2-one (C70) at 30.60% in *A. chinensis* pollen, 1-pentanol (C12) at 22.56% in *E. manipuliflora* pollen, nonanal (C111) at 19.31% in *C. intybus* pollen, 3-hexen-1-ol (C33) at 17.59% in *Ranunculus* sp. pollen, 1-hexanol (C40) at 15.77% in *Trifolium* sp. pollen, and 1,2-dimethylbenzene (C37) at 13.32% in *Rubus* sp. pollen.

In terms of chemical classes, the highest number of hydrocarbons (*n* = 25) was detected in *E. manipuliflora* and *P. rhoeas* pollen, while the lowest number was found in *Cistus* sp. pollen (*n* = 4) and *Ranunculus* sp. pollen (*n* = 6). Among the group of hydrocarbons, octane (C23) and o-xylene (C37) were detected at notably high levels in certain unifloral pollen types, such as *C. sativa*, *Acer* sp., *Lamium* sp., *Rubus* sp., and *C. intybus*. As previously mentioned, certain hydrocarbons were exclusively found in specific species, likely contributing to the attractiveness of their pollen.

The highest number of aldehydes was found in the pollen of *A. chinensis* (*n* = 11) and (*Ranunculus* sp.) (*n* = 11). Nonanal (C111) stood out both for its presence in all tested species and for the exceptionally high concentrations found in *C. intybus* pollen (19.31%), *Acer* sp. pollen (16.98%), *Trifolium* sp. pollen (13.84%), *Cistus* sp. pollen (11.43%), *C. sativa* pollen (10.4%), *P. rhoeas* pollen (8.82%), and *A. chinensis* pollen (8.36%). In contrast to the findings of Kaškoniene et al. [40], who report the presence of hexanal (C24) in 92.9% of mixed pollen samples, in the present study, it was detected in only 5 out of 16 unifloral pollen types, where it was the dominant volatile compound in *P. inserta* pollen, accounting for 47.16% of the total volatiles. The lowest number of aldehydes (*n* = 5) was found in *P. halepensis* pollen, and their total percentage did not exceed 3% of the total volatiles.

Among alcohols, 1-penten-3-ol (C4), 1-hexanol (C40), and 2-ethyl-1-hexanol (C85) were detected in the majority of samples. The highest number of alcohol-containing compounds was found in *Ranunculus* sp. pollen (*n* = 9), while the lowest was in *Lamium* sp. pollen (*n* = 2). Notably, 4-ethyl-5-methylthiazole (C102) was detected in exceptionally high concentrations in *B. napus* pollen (54.52%), 2-ethyl-1-hexanol (C85) in *Cistus* sp. pollen (39.96%), 1-pentanol (C12) in *E. manipuliflora* pollen (22.56%), 3-hexen-1-ol (C33) in *Ranunculus* sp. pollen (17.59%), and 1-hexanol (C40) in *Trifolium* sp. pollen (15.77%).

The highest number of esters was detected in *P. rhoeas* pollen (*n* = 5), followed by *E. manipuliflora* pollen (*n* = 4) and *S. irio* pollen (*n* = 4). In 50% of the tested pollen samples, no esters were detected. In *E. manipuliflora*, the ethyl ester of propanoic acid (C10) and the 2-methyl-ethyl ester of butanoic acid (C30) stood out, with concentrations of 3.83% and 4.53%, respectively. In *P. rhoeas* and *S. irio*, the percentage of esters was lower than 1%.

Among ketones, the highest number was detected in *A. chinensis* pollen (*n* = 5), *Cistus* sp. pollen (*n* = 5), and *Verbascum* sp. pollen (*n* = 5), while they were absent from *Acer* sp. and *P. halepensis* pollen. The percentage of 6-methyl-5-hepten-2-one (C70) exceeded 8% in *Rubus* sp. and *Trifolium* sp. pollen and was the dominant compound in *A. chinensis* pollen (30.6%) and *Verbascum* sp. pollen (61.99%).

Finally, from the acid chemical class, only two VOCs (C80, C125) were detected, both in *Cistus* sp. pollen. Among heterocyclic compounds, 2-ethylfuran (C7) stood out for its high percentage in *Lamium* sp. pollen (18.91%), *Rubus* sp. pollen (8.13%), and *Trifolium* sp. pollen (8.35%). Lastly, in the other VOC chemical class, the presence of 4-isothiocyanato-1-butyne (C71) was particularly notable in the Brassicaceae family species.

## 4. Discussion

Bee pollen contains a wide array of VOCs, among which hydrocarbons, aldehydes, ketones, and esters are predominant [22,25,42,44,49,50,51]. In the present study, hydrocarbons represented the most abundant chemical group, accounting for 32.9% of the total volatile profile, followed by alcohols (17.9%), aldehydes (17.1%), and ketones (10.7%). The predominance of hydrocarbons at levels exceeding 25% of total volatile compounds has also been reported by Dobson and Bergström [25], who analyzed unifloral pollen samples from species of the Asteraceae family. Similarly, a hydrocarbon content greater than 25% has been documented in the pollen of *P. rhoeas* [22] and *Lycopersicon esculentum* [25]. In contrast, Flamini et al. [23], studying the volatile characteristics of *Citrus limon* pollen from Italy, highlighted terpenes as the dominant constituents. Regarding alcohols, their relative abundance has been reported to range from 10% to 25% in species such as *Sonchus arvensis*, *Lonicera caprifolium* [25], *Ranunculus acris* [36], and several members of the Rosaceae family [11,22,27]. Aldehydes typically represent less than 10% of the total volatiles [22,25], with the exception of certain Rosaceae species, which exhibit a higher aldehyde content in their pollen [11,27]. Conversely, Karabagias et al. [42] reported that aldehydes were the dominant volatiles in mixed bee pollen samples from Northwestern Greece. Moreover, Csóka et al. [38], analyzing the volatile profiles of 14 unifloral pollen samples, observed notable proportions of alcohols (6.21–15.10%), aldehydes (0.83–25.30%), and ketones (0.26–22.72%).

The botanical origin appears to play a decisive role in shaping the volatile profile of pollen. Indeed, both quantitative and qualitative differences in total VOCs were observed among the different unifloral pollen samples analyzed. Among the 16 unifloral samples examined, *E. manipuliflora*, *P. rhoeas*, and *S. irio* stood out due to the high number of VOCs detected—54, 51, and 42 compounds, respectively. The notably rich volatile profile of *E. manipuliflora* may be linked to the particular foraging preference honey bees exhibit for its pollen, despite its relatively low crude protein (16.26% D.M. [Dry Matter]) and total lipid (0.4% D.M.) content [52,53]. It is also worth noting that the flowering period of *E. manipuliflora* coincides with autumn, a time when pollen sources are scarce and protein availability is limited for pollinators [52]. As for *S. irio* and *P. rhoeas*, these wild species are abundant in the study region, and their pollen is collected in large quantities by honey bees during spring [33,47,52]. Moreover, the strong foraging preference of honey bees for Brassicaceae species has been reported by other researchers as well [54,55]. Csóka et al. [38] highlighted the high number of aroma-active zones in the pollen of *B. napus*, *P. rhoeas*, and *Rubus fruticosus*, with *P. rhoeas* showing the most diverse VOC profile among the 14 unifloral samples examined. In contrast to the present findings, Dobson et al. [22] reported a smaller number of VOCs in *P. rhoeas* pollen, detecting only 33 compounds, while Bergström et al. [36] identified just 5 VOCs in the unifloral pollen of *Ranunculus acris*. In the current study, the lowest number of VOCs was detected in *Pinus* sp. (pine) and *Lamium* sp. pollen, with only 25 compounds identified. Von Aufsess [56], comparing volatile profiles of anemophilous and entomophilous species—mainly Poaceae—highlighted the lack of pronounced odor in the pollen of wind-pollinated species and the compositional differences in their VOC profiles. This trend is confirmed by Dobson & Bergström [25], who studied four anemophilous species (*Pinus sylvestris*, *Betula verrucosa*, *Quercus robur*, and *Dactylis glomerata*), all of which showed reduced VOC diversity.

Among the exclusive organic compounds detected in the pollen of the examined plant species, the highest number of unique compounds was identified in *P. rhoeas* (*n* = 6), *Cistus* sp. (*n* = 6), and *E. manipuliflora* (*n* = 6), followed by *A. chinensis* (*n* = 5) and *P. halepensis* (*n* = 5). Although most of these characteristic compounds were present in relatively low percentages (Table 2), a more in-depth investigation into some of these unique metabolites appears to be of particular interest. Notably, 4–methyl–5–nonanone (C113), found exclusively in the pollen of *E. manipuliflora* at a concentration of 6.11%, has been reported as a beetle-attractant pheromone with practical applications in insect trapping [57,58]. Therefore, the presence of such an attractant compound in Erica pollen may partially explain the notable preference exhibited by honey bees toward this species. Similarly, the occurrence of geraniol (C128) in *P. rhoeas* pollen (4.04%) could be associated with its attractiveness. Hohmann [59] demonstrated that the addition of various aromatic compounds, including geraniol, to cellulose powder significantly increased its uptake by honey bees. Furthermore, one of the best-known orientation pheromones in honey bees—produced by the Nasonov gland and functioning as an attractant for worker honey bees—is composed of a mixture of compounds that includes geraniol [60,61,62,63]. Among the unique compounds detected in the pollen of *Cistus* sp., caprylic acid (C125) was found in the highest concentration. Saa-Otero et al. [64], in their study of fatty acid composition in eight unifloral pollen types, reported the presence of caprylic acid in trace amounts in other pollen taxa such as *C. sativa*, *Erica*, *Eucalyptus*, *Rubus*, *Quercus robur*, and *Halimium alyssoides*. In *A. chinensis*, geranial (C97) and neral (C93)—the two stereoisomers that comprise citral—were detected in small amounts. Citral is another attractant pheromone also found in the Nasonov gland secretion [60,61,62,63]. However, Blum [65], studying the reaction of *Trigona subterranea* worker bees to high concentrations of citral, reported aggressive behavior and in some cases nest abandonment for the duration of exposure, indicating a potential concentration-dependent behavioral response. In *P. halepensis*, the exclusive compounds β-myrcene (C74) and δ-3-carene (C77) were particularly abundant, detected at 14.03% and 10.47%, respectively. These two terpenes are known constituents of several essential oils with documented antifungal properties [66,67,68,69]. Llusià & Peñuelas [70] linked terpene emission in pine seedlings to environmental conditions, particularly temperature and humidity. The high concentrations of these terpenes in *P. halepensis* are consistent with findings by Blanch et al. [71], who reported elevated levels of β-myrcene, δ-3-carene, and α- and β-pinene in the foliage of this species.

The exclusive occurrence of isothiocyanate compounds (C39, C44, C71) in species belonging to the Brassicaceae family is worth further discussion. However, due to their detection in the pollen of both *S. irio* and *B. napus*, they were not included among the species-specific compounds in Table 2. Isothiocyanates and thiocyanates are degradation products of glucosinolates following their hydrolysis by the enzyme myrosinase [72,73]. These compounds are largely responsible for the characteristic odors of cruciferous plants, which have been shown to act either as attractants [74] or repellents [75] for certain pollinators. The strong presence of isothiocyanates in Brassicaceae species has also been reported by other researchers [38,74,76]. Additionally, Tollsten & Bergström [77] noted the emission of isothiocyanates in crucifers upon the mechanical injury of their tissues. Regarding the pollinators’ response to these compounds, it has been demonstrated that certain insects, including bees, are capable of detecting thiocyanates through their antennae [78,79].

## 5. Conclusions

Among the analyzed unifloral pollen species, both quantitative and qualitative differences and similarities in their volatile compound profiles were observed. Notably, the pollen of *E. manipuliflora*, *P. rhoeas*, and *S. irio* exhibited a significantly higher number of VOCs compared to other species. A total of 54 VOCs were identified in *E. manipuliflora*, 51 in *P. rhoeas*, and 42 in *S. irio*. The presence of specific VOCs in certain pollen species may be associated with their increased attractiveness to honey bees. For instance, 4-methyl-5-enal was detected exclusively in *E. manipuliflora* pollen, while isothiocyanate compounds were uniquely found in species of the Brassicaceae family. Thus, when assessing the apicultural value of a pollen-producing plant, it is crucial to consider not only its basic chemical composition and nutritional value but also its volatile compound profile, as these compounds significantly influence the plant’s attractiveness to honey bees and its pollen collection efficiency. Moreover, the characteristic volatile compounds identified in the 16 examined taxa could potentially serve as reliable indicators for determining botanical origin, pending further studies on other unifloral pollen species to confirm the uniqueness of their presence in specific pollinating plants.

## Figures and Tables

**Table 1:** Coding and identification of VOCs detected in the 16 unifloral pollen samples, along with their major fragment ions. Compounds are listed in ascending order of retention time (RT).

α/α	Code	Volatile Compound	RT (min)
1	C1	Methyl propanoate (*m*/*z* 57, 88)	2.61
2	C2	isomer of methylbutanal (*m*/*z* 58, 71, 86)	2.78
3	C3	Benzene (*m*/*z* 51, 58, 78)	2.95
4	C4	1-Penten-3-ol (*m*/*z* 55, 57)	3.09
5	C5	Heptane (*m*/*z* 57, 71, 100)	3.30
6	C6	4-Methylcyclohexanol (*m*/*z* 57, 81, 96)	3.33
7	C7	2-ethylfuran (*m*/*z* 53, 81, 96)	3.37
8	C8	2-Hexenal (*m*/*z* 55, 69, 83, 90)	3.41
9	C9	Unknown (*m*/*z* 59, 71, 85)	3.53
10	C10	Ethyl propionate (*m*/*z* 57, 75, 102)	3.56
11	C11	2-Ethyl-2-butenal (*m*/*z* 55, 69, 83, 98)	3.97
12	C12	1-Pentanol (*m*/*z* 55, 70)	4.00
13	C13	Isothiocyanatomethane (*m*/*z* 78)	4.21
14	C14	Dimethyl disulfide (*m*/*z* 61, 79, 94)	4.23
15	C15	3-Methyl-2-butenal (*m*/*z* 55, 83, 94)	4.51
16	C16	2-Methylpropanoic acid ethyl ester (*m*/*z* 71, 88, 116)	4.61
17	C17	Methyl-benzene {Toleune} (*m*/*z* 51, 65, 91)	4.75
18	C18	2-Penten-1-ol (*m*/*z* 57, 68, 70, 91)	4.83
19	C19	Butanoic acid, 2-methyl-, methyl ester (*m*/*z* 57, 88, 101)	5.15
20	C20	1-Hectene (*m*/*z* 55, 70, 83, 112)	5.43
21	C21	Unknown (m/s 55, 67, 85, 97)	5.44
22	C22	3-Methyl-3-cyclohexen-1-ol (*m*/*z* 55, 60, 69, 84, 97)	5.48
23	C23	Octane (*m*/*z* 57, 71, 85, 114)	5.73
24	C24	Hexanal (*m*/*z* 56, 67, 71, 85)	5.87
25	C25	2-Octene (*m*/*z* 55, 70, 83, 112)	6.07
26	C26	3-Methyl-1,4-heptadiene (*m*/*z* 55, 68, 79, 81, 95, 110)	6.29
27	C27	3-Octene (*m*/*z* 55, 70, 81, 95, 112)	6.39
28	C28	Furfural (*m*/*z* 67, 96)	7.31
29	C29	2-Methyl-pentan-1-ol (*m*/*z* 56, 69, 84)	7.95
30	C30	Ethyl 2-methylbutyrate (*m*/*z* 57, 74, 85, 102, 115)	8.25
31	C31	2-Hexenal (*m*/*z* 55, 69, 83, 98)	8.35
32	C32	Ethyl 3-methylbutyrate (*m*/*z* 55, 57, 69, 83, 85, 88)	8.48
33	C33	3-hexen-1-ol (*m*/*z* 67, 77, 82, 91, 106)	8.57
34	C34	Ethylbenzene (*m*/*z* 67, 91, 106)	8.69
35	C35	5-cyano-1-pentene (*m*/*z* 55, 67, 80, 94)	8.71
36	C36	2-Furanmethanol (*m*/*z* 53, 69, 81, 98)	8.71
37	C37	1,2-Xylene (*m*/*z* 91, 106)	9.12
38	C38	2-Hexen-1-ol (*m*/*z* 57, 67, 82)	9.23
39	C39	Isomer of allyl isothiocyanate (*m*/*z* 58, 72, 99)	9.43
40	C40	1-Hexanol (*m*/*z* 56, 69)	9.44
41	C41	3-Methyl-1-butanol acetate (*m*/*z* 55, 70, 87)	9.86
42	C42	2-Methyl-1-butanol acetate (*m*/*z* 55, 70)	10.04
43	C43	2-Cyclopentene-1,4-dione (*m*/*z* 54, 68, 96)	10.21
44	C44	Isomer of allyl isothiocyanate (*m*/*z* 72, 99)	10.23
45	C45	Styrene (*m*/*z* 51, 78, 104)	10.47
46	C46	1,4-Xylene (*m*/*z* 91, 106)	10.56
47	C47	2-Heptanone (*m*/*z* 58, 71, 81, 99, 114)	10.75
48	C48	Nonane (*m*/*z* 57, 71, 85, 114)	11.28
49	C49	Heptanal (*m*/*z* 55, 70, 81, 96)	11.43
50	C50	1-(Furan-2-yl)ethanol (*m*/*z* 60, 73, 95, 110)	12.14
51	C51	Methyl hexanoate (*m*/*z* 59, 74, 87, 99)	13.23
52	C52	2,6,6-Trimethylbicyclo[3.1.1]hept-2-ene {a-pinene} (*m*/*z* 77, 79, 91, 93, 105, 121, 136)	13.59
53	C53	2,2-Dimethyl-3-methylidenebicyclo[2.2.1]heptane {Camphene} (*m*/*z* 67, 79, 93, 107, 121, 136)	14.51
54	C54	Unknown (*m*/*z* 53, 67, 81, 95, 124)	14.75
55	C55	1-Acetyl-1,2-epoxy-cyclopentane (*m*/*z* 55, 83, 97, 111)	14.94
56	C56	Propylbenzene (*m*/*z* 65, 91, 120)	15.35
57	C57	4,4-Dimethylcyclohex-2-en-1-one (*m*/*z* 57, 72, 111, 126)	15.74
58	C58	2-(5-ethenyl-5-methyloxolan-2-yl)propanal (Lilac aldehyde) (*m*/*z* 55, 67, 77, 93, 105, 139)	15.76
59	C59	6-Methylheptan-2-one (*m*/*z* 58, 71, 95, 110)	15.91
60	C60	Benzaldehyde (*m*/*z* 51, 77, 106)	15.98
61	C61	1-Ethyl-2-methylbenzene (*m*/*z* 77, 91, 105, 120)	16.11
62	C62	5-Methyl-2-furancarbaldehyde (*m*/*z* 53, 81, 110, 120)	16.59
63	C63	Trimethylbenzene (*m*/*z* 77, 91, 105, 120)	16.72
64	C64	6,6-dimethyl-2-methylidenebicyclo[3.1.1]heptane {b-pinene}(*m*/*z* 69, 77, 79, 91, 93, 106, 121, 136)	17.22
65	C65	3-Methylbutyl propionate (*m*/*z* 57, 70, 75)	17.49
66	C66	1-Heptanol (*m*/*z* 56, 70)	17.63
67	C67	1-Ethyl-3-methylbenzene (*m*/*z* 57, 70, 105, 120)	17.75
68	C68	1-Octen-3-ol (*m*/*z* 57, 72, 85, 110)	18.19
69	C69	6-Octen-2-one (*m*/*z* 55, 68, 97, 108, 126)	18.61
70	C70	6-Methylhept-5-en-2-one (*m*/*z* 55, 69, 83, 93, 108, 126)	18.84
71	C71	4-Isothiocyanato-1-butene (*m*/*z* 55, 72, 85, 113)	18.87
72	C72	1,2,3-Trimethylbenzene (*m*/*z* 77, 91, 105, 120)	19.20
73	C73	2-Pentylfuran (*m*/*z* 53, 81, 138)	19.34
74	C74	7-methyl-3-methylideneocta-1,6-diene (beta-myrcene) (*m*/*z* 69, 79, 93)	19.42
75	C75	6-Methylhept-5-en-2-ol (*m*/*z* 41, 55, 69, 95)	19.78
76	C76	Decane (*m*/*z* 57, 71, 81, 113, 142)	20.32
77	C77	3,7,7-Trimethylbicyclo[4.1.0]hept-3-ene {δ-3-Carene} (*m*/*z* 77, 93, 105, 121, 136)	20.50
78	C78	Octanal (*m*/*z* 57, 69, 84, 95, 100, 146)	20.63
79	C79	Isomer of Heptadienal (*m*/*z* 53, 67, 81, 110, 281)	21.33
80	C80	Hexanoic acid (*m*/*z* 60, 73, 87)	21.65
81	C81	1,2,4-trimethylbenzene (*m*/*z* 77, 105, 120)	21.78
82	C82	1-methyl-4-propan-2-ylbenzene {p-cymene} (*m*/*z* 77, 91, 119, 134)	22.07
83	C83	1-methyl-4-prop-1-en-2-ylcyclohexene {limonene} (*m*/*z* 53, 68, 93, 107, 136)	22.31
84	C84	2,3-dihydro-1H-indene {Indane} (*m*/*z* 91, 115, 117)	22.82
85	C85	2-Ethylhexan-1-ol (*m*/*z* 57, 70, 83, 98)	23.29
86	C86	3,5,5-trimethylcyclohex-3-en-1-one {beta-Isophorone} (*m*/*z* 55, 67, 81, 96, 123, 138)	23.81
87	C87	3,7-dimethylocta-1,3,6-triene {Ocimene} (*m*/*z* 53, 65, 80, 93, 105, 120, 136)	24.45
88	C88	1-methyl-2-propylbenzene (*m*/*z* 77, 91, 105, 134)	24.53
89	C89	Butylbenzene (*m*/*z* 91, 105, 119, 134)	24.86
90	C90	1-methyl-4-propan-2-ylcyclohexa-1,4-diene {gamma-Terpinene} (*m*/*z* 65, 77, 93, 119, 121, 81, 94, 111, 137)	24.98
91	C91	1-methyl-2-propan-2-ylbenzene {cymene} (*m*/*z* 91, 119, 134)	25.13
92	C92	1-methyl-3-propylbenzene (*m*/*z* 77, 105, 134)	25.64
93	C93	(2Z)-3,7-dimethylocta-2,6-dien-1-ol {beta Citral, nerol} (*m*/*z* 55, 69, 82, 91, 109, 123)	25.85
94	C94	isomer of Octadien-2-one (*m*/*z* 53, 81, 95, 109, 124)	26.41
95	C95	isomer of Cymene (*m*/*z* 55, 81, 95, 119, 134)	26.54
96	C96	1-Octanol (*m*/*z* 56, 70, 84)	26.64
97	C97	(2E)-3,7-dimethylocta-2,6-dienal {alpha-Citral} (*m*/*z* 55, 69, 91, 109, 123)	26.79
98	C98	Furan-2,5-dicarboxylic acid (*m*/*z* 95, 124)	26.97
99	C99	3,5-Dihydroxytoleune {Methylresorcinol} (*m*/*z* 55, 69, 95, 124)	26.99
100	C100	3,7-dimethylocta-4,6-dien-3-ol (*m*/*z* 55, 69, 95, 109, 124, 134)	27.01
101	C101	1-ethyl-2-methylbenzene {Cymene} (*m*/*z* 91, 119, 134)	27.01
102	C102	4-Ethyl-5-methyl-1, 3-thiazole (*m*/*z* 53, 55, 67, 72, 85, 99, 126)	27.09
103	C103	1-methyl-4-propan-2-ylidenecyclohexene {Terpinolene} (*m*/*z* 79, 93, 105, 121, 136)	27.12
104	C104	Unknown (*m*/*z* 57, 70, 83, 124)	27.16
105	C105	2-Nonanone (*m*/*z* 58, 71, 95, 124)	27.96
106	C106	5-methyl-5-octen-2-ol (*m*/*z* 58, 71, 81, 95, 124, 142)	27.97
107	C107	Isomer of octadien-2-one (*m*/*z* 58, 81, 95, 109, 124)	27.98
108	C108	Undecane (*m*/*z* 57, 71, 85)	28.28
109	C109	Ethyl heptanoate (*m*/*z* 55, 60, 70, 88, 101, 113)	28.34
110	C110	1-ethyl-2-methylbenzene {Cymene} (*m*/*z* 57, 71, 119, 134)	28.41
111	C111	Nonanal (*m*/*z* 57, 70, 82, 98, 114)	28.71
112	C112	1,2,3,4-tetramethylbenzene (*m*/*z* 91, 119, 134)	29.03
113	C113	4-methylnonan-5-one (*m*/*z* 57, 71, 85, 99, 114)	29.34
114	C114	3,5,5-trimethylcyclohex-3-en-1-one {beta-Isophorone} (*m*/*z* 54, 82, 95, 138)	29.37
115	C115	methyl octanoate (*m*/*z* 55, 74, 87, 115, 148)	30.18
116	C116	2-methyl-2,3-dihydro-1H-indene (*m*/*z* 51, 64, 91, 117, 132)	30.39
117	C117	3,5-dihydroxy-6-methyl-2,3-dihydropyran-4-one (*m*/*z* 55, 72, 101, 144)	31.32
118	C118	Unknown (*m*/*z* 55, 70, 85, 144)	31.77
119	C119	1-isothiocyanato-4-methylpentane (*m*/*z* 56, 69, 72, 110, 128, 143)	32.06
120	C120	(1R,2S,4R)-1,7,7-trimethylbicyclo[2.2.1]heptan-2-ol {borneol} (*m*/*z* 69, 79, 95, 110)	32.09
121	C121	2-Nonanal (*m*/*z* 55, 70, 83, 96)	32.11
122	C122	Ethyl benzoate (*m*/*z* 77, 105, 122, 150)	32.50
123	C123	Naphthalene (*m*/*z* 102, 128)	32.88
124	C124	Nonanol (*m*/*z* 56, 69, 83, 98)	33.01
125	C125	Octanoic acid (Caprylic acid) (*m*/*z* 60, 73, 85, 101)	34.08
126	C126	Decanal (*m*/*z* 57, 70, 82, 95, 112)	34.73
127	C127	2,6,6-trimethylcyclohexene-1-carbaldehyde {beta-cyclocitral} (*m*/*z* 67, 81, 91, 109, 123, 137, 152)	35.28
128	C128	(2Z)-3,7-dimethylocta-2,6-dien-1-ol {geraniol} (*m*/*z* 69, 93, 121)	35.97
129	C129	(Z)-3,7-dimethylocta-2,6-dienal {isomer of citral} (*m*/*z* 69, 84, 94, 109, 134)	36.53
130	C130	5-butyloxolan-2-one {gamma-octalactone} (*m*/*z* 85, 100)	37.44
131	C131	2-Decenal (*m*/*z* 55, 70, 85, 98, 110)	37.59
132	C132	(Z)-3,7-dimethylocta-2,6-dienal {isomer of citral} (*m*/*z* 69, 84, 94, 109, 134)	38.07
133	C133	Decatriene (*m*/*z* 57, 71, 85)	39.50
134	C134	(2E)-3,7-dimethylocta-2,6-dien-1-ol{geraniol} (*m*/*z* 69, 79, 93, 121, 136)	42.43
135	C135	6,10-dimethylundeca-5,9-dien-2-one (*m*/*z* 69, 93, 107, 125, 136, 151)	44.92
136	C136	H/C (*m*/*z* 57, 71, 85, 99, 113, 141, 183)	45.11
137	C137	(1R,4E,9S)-4,11,11-trimethyl-8-methylidenebicyclo[7.2.0]undec-4-ene {caryophyllene} (*m*/*z* 69, 79, 93, 105, 120, 133, 147, 161, 189)	45.20
138	C138	(3S,4aR,8aS)-8a-methyl-5-methylidene-3-prop-1-en-2-yl-1,2,3,4,4a,6,7,8-octahydronaphthalene {(−)-beta-selinene} (*m*/*z* 53, 67, 79, 91, 95, 105, 119, 133, 147, 161, 175, 189, 204)	45.47
139	C139	Decapentaene (*m*/*z* 57,71,85,99)	45.82
140	C140	(3E,6E)-3,7,11-trimethyldodeca-1,3,6,10-tetraene {alpha-Farnesene} (*m*/*z* 55, 69, 79, 93, 107, 119, 123)	45.99

In parentheses, the mass spectrum fragments of each compound are described, and the main fragment is highlighted with underlining.

**Table 2:** Compounds exclusively detected in each of the unifloral pollen samples and their percentage contribution to the total volatile profile.

Pollen Species	Unique Compounds and Their Percentage Participation
*Papaver rhoeas*	C89 (0.61%), C112 (1.23%), C116 (0.98%), C128 (4.04%), C129 (2.44%), C134 (1.03%)
*Cistus* sp.	C80 (1.08%), C96 (1.97%), C105 (1.07%), C125 (4.01%), C130 (0.75%), C131 (0.44%)
*Pinus halepensis*	C53 (1.32%), C74 (14.03%), C77 (10.47%), C103 (1.11%), C122 (0.31%)
*Actinidia chinensis*	C69 (2.36%), C93 (0.57%), C97 (2.07%), C109 (0.53%), C140 (0.65%),
*Verbascum* sp.	C8 (1.59%), C59 (2.04%), C114 (5.91%), C127 (1.08%)
*Erica manipuliflora*	C19 (0.89%), C41 (5.62%), C42 (1.15%), C113 (6.11%),
*Trifolium* sp.	C106 (0.76%), C121 (1.22%)
*Ranunculus* sp.	C43 (5.8%), C98 (0.76%)
*Cichorium intybus*	C1 (0.76%), C13 (3.01%)
*Brassica napus*	C35 (3.49%)
*Parthenocissus inserta*	C133 (0.95%)
*Lamium* sp.	C138 (3.02%)

**Table 3:** Main volatile compounds identified in each of the examined unifloral pollen samples.

*Acer* sp.	*Actinidia chinensis*	*Brassica napus*	*Castanea sativa*
C23 (26.79%)	C70 (30.60%)	C102 (54.52%)	C23 (67.90%)
C111 (16.98%)	C49 (9.93%)	C71 (11.33%)	C111 (10.4%)
C29 (4.06%)	C111 (8.36%)	C49 (8.02%)	C40 (3.16%)
***Cichorium intybus***	***Cistus* sp.**	***Erica manipuliflora***	***Lamium* sp.**
C111 (19.31%)	C85 (39.96%)	C12 (22.56%)	C23 (29.31%)
C37 (16.08%)	C111 (11.43%)	C45 (9.14%)	C7 (18.91%)
C23 (10.26%)	C28 (10.37%)	C113 (6.11%)	C3 (9.18%)
***Parthenocissus inserta***	***Papaver rhoeas***	***Pinus halepensis***	***Ranunculus* sp**.
C24 (47.16%)	C72 (9.43%)	C64 (38.42%)	C33 (17.59%)
C37 (6.83%)	C37 (9.07%)	C52 (14.87%)	C4 (14.98%)
C111 (5.23%)	C111 (8.82%)	C74 (14.03%)	C31 (13.9%)
***Rubus* sp**.	***Sisymbrium irio***	***Trifolium* sp.**	***Verbascum* sp.**
C37 (13.32%)	C71 (58.45%)	C40 (15.77%)	C70 (61.99%)
C17 (9.99%)	C23 (5.51%)	C23 (15.55%)	C114 (5.91%)
C7 (8.13%)	C44 (3.92%)	C70 (9.88%)	C37 (2.93%)

## Data Availability

The original contributions presented in the study are included in the article; further inquiries can be directed to the corresponding author. Data supporting the reported results are stored at the Laboratory of Apiculture-Sericulture, AUTH.
